# MicroRNA Genes and Their Target 3′-Untranslated Regions Are Infrequently Somatically Mutated in Ovarian Cancers

**DOI:** 10.1371/journal.pone.0035805

**Published:** 2012-04-20

**Authors:** Georgina L. Ryland, Jennifer L. Bearfoot, Maria A. Doyle, Samantha E. Boyle, David Y. H. Choong, Simone M. Rowley, Australian Ovarian Cancer Study Group, Richard W. Tothill, Kylie L. Gorringe, Ian G. Campbell

**Affiliations:** 1 Victorian Breast Cancer Research Consortium Cancer Genetics Laboratory, Peter MacCallum Cancer Centre, East Melbourne, Victoria, Australia; 2 Centre for Cancer Research, Monash Institute of Medical Research, Monash University, Clayton, Victoria, Australia; 3 Department of Pathology, University of Melbourne, Parkville, Victoria, Australia; 4 Bioinformatics Core Facility, Peter MacCallum Cancer Centre, East Melbourne, Victoria, Australia; 5 Peter MacCallum Cancer Centre, East Melbourne, Victoria, Australia; 6 Molecular Genomics Core Facility, Peter MacCallum Cancer Centre, East Melbourne, Victoria, Australia; Baylor College of Medicine, United States of America

## Abstract

MicroRNAs are key regulators of gene expression and have been shown to have altered expression in a variety of cancer types, including epithelial ovarian cancer. MiRNA function is most often achieved through binding to the 3′-untranslated region of the target protein coding gene. Mutation screening using massively-parallel sequencing of 712 miRNA genes in 86 ovarian cancer cases identified only 5 mutated miRNA genes, each in a different case. One mutation was located in the mature miRNA, and three mutations were predicted to alter the secondary structure of the miRNA transcript. Screening of the 3′-untranslated region of 18 candidate cancer genes identified one mutation in each of *AKT2*, *EGFR*, *ERRB2* and *CTNNB1*. The functional effect of these mutations is unclear, as expression data available for *AKT2* and *EGFR* showed no increase in gene transcript. Mutations in miRNA genes and 3′-untranslated regions are thus uncommon in ovarian cancer.

## Introduction

MicroRNAs (miRNAs), a class of small non-coding RNA molecules, have important regulatory roles in diverse cellular pathways including proliferation, differentiation, senescence and metabolism [1]. This regulation is achieved through semi-complementary base paring with the 3′-untranslated region (3′-UTR) of the target messenger RNA (mRNA) [1]–[3], as well as the 5′-untranslated region or coding regions of mRNAs, which are subsequently degraded or post-transcriptionally silenced [4]–[6]. Accumulating evidence now demonstrates that miRNA expression is aberrant in cancer [7], leading to the hypothesis that alterations in miRNA pathways may be an important step in the initiation and progression to malignancy. Consistent with this hypothesis is the observation that miRNA genes are frequently localised in genomic regions commonly altered in cancer, including minimal regions of deletion, loss of heterozygosity and amplification as well as fragile sites [8]–[10]. Mutation is an alternative mechanism for miRNA deregulation in the cancer setting, whereby mutation may alter miRNA transcription, processing or miRNA-mRNA interactions. This mechanism was first described by Calin *et al.* [8], who identified a germline variation in *hsa-miR-16-1* that was linked with susceptibility to chronic lymphocytic leukaemia. Since then, germline polymorphisms in miRNA genes have been associated with predisposition to other cancer types [11]. Despite the hypothesis that miRNAs may function as conventional oncogenes or tumor suppressors, several studies have suggested that somatic mutation within miRNAs are a rare occurrence and those that have been reported show little effect on miRNA activity [10], [12]–[14]. However, the majority of these studies have favoured a candidate gene approach and to date, un-biased assessment of the occurrence of somatic alterations in miRNA genes in any cancer type is lacking.

Analogous to mutations occurring within miRNA seed regions, mutations occurring within the target mRNA sequence may alter binding and subsequent miRNA-dependent regulation of gene expression. While germline alterations at miRNA binding sites in 3′-UTRs may contribute to cancer susceptibility [11], [15]–[17], reports of somatic mutations occurring in a similar context is limited to a single case report [13] and has yet be to investigated in large tumor cohorts. If somatic mutations do occur at miRNA binding sites, it would constitute a previously unexplored genetic mechanism for repression or activation of a cancer-associated mRNA.

Aberrant miRNA activity is frequently associated with the pathogenesis and progression of epithelial ovarian cancer, the most common form of ovarian malignancy. MiRNA profiling studies consistently observe global silencing of miRNA expression in ovarian tumors, which is contributed to in part by genomic loss and epigenetic alterations [10], [18]–[22]. Similarly, expression of many known and putative cancer genes is dysregulated in ovarian cancer, for example *BRCA2*, for which only a proportion of the observed loss of expression can be attributed to mutation, and promoter methylation is not observed [23]. Previously, we have demonstrated that the frequency of somatic mutations in 10 cancer-implicated miRNAs is low in ovarian tumors [24]. In the present study, we extend this analysis by comprehensively characterising somatic mutations in 712 miRNA genes using massively parallel targeted re-sequencing. In addition, we screened the 3′-UTRs of 18 candidate cancer genes with the aim of identifying somatic mutations that alter predicted miRNA binding sites. Although these genes are frequently implicated in the tumorigenic process, coding mutations, methylation or copy number alterations only account for a subset of the expression differences seen in ovarian tumors.

## Results and Discussion

### Somatic mutations targeting microRNA genes are infrequent events in ovarian tumors

**Figure 1:**
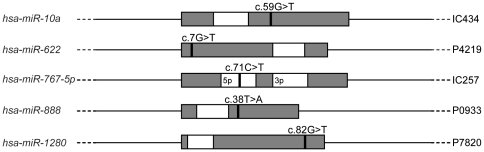
Somatic mutations identified in microRNA genes. Tumor specific mutations are marked as black bars relative to the mature microRNA (white box) and precursor microRNA (grey box) sequences. The positions of mutations are reported with respect to the following precursor microRNA transcripts: *hsa-miR-10a* NR_029608.1; *hsa-miR-622* NR_030754.1; *hsa-miR-767-5p* NR_030409.1; *hsa-miR-888* NR_030592.1; *hsa-miR-1280* NR_031703.1.

To investigate whether mutations in miRNA genes contribute to altered miRNA activity in ovarian cancer, 86 primary epithelial ovarian tumors were assessed for somatic mutations in genomic regions corresponding to precursor or mature miRNA sequences. Clinical characteristics of these cases are summarised in Table S1. Targeted next generation sequencing was used to assess 712 miRNA genes annotated in the Sanger miRNA database (version 13.0, March 2009). Following data alignment, 95% of targeted bases within the 712 miRNA genes had a minimum 10-fold sequence coverage, with a corresponding mean coverage of 92-fold. Filtering to remove germline variants detected in matched normal lymphocyte DNA and validation by conventional sequencing identified somatic mutations in 5 miRNA genes: *hsa-miR-10a*, *hsa-miR-622*, *hsa-miR-767-5p*, *hsa-miR-888* and *hsa-miR-1280* (Figure 1). Overall, somatic mutations were detected in 6% (5/86) of tumors and in less than 1% (5/712) of miRNA genes analysed, with no miRNA genes recurrently targeted by mutation. Consistent with previous reports, mutations within mature miRNAs were uncommon; only one mutation was located within the mature region of *hsa-miR-767-5p* (but external to the seed region), whilst the remaining four occurred within the precursor hairpin. This data is in agreement with previous smaller scale studies suggesting that somatic mutations in miRNA genes are an infrequent event in tumor samples [8], [12], [14], [24], [25].

MiRNA biogenesis is a multistep process initiated by RNA polymerase II-mediated transcription, followed by RNAse III-dependent trimming into a hairpin intermediate and subsequent cleavage into a functional mature miRNA [1], [26]. This process is dependent on sequence motifs and RNA secondary structure elements within the primary and precursor miRNA molecules [26]. As such, mutations arising in precursor regions may alter RNA secondary structure and thereby block processing into mature miRNA. To determine if RNA structural changes may result from the somatic miRNA mutations identified, we used the RNAfold program [27] to predict the most stable secondary structure for both the wild-type and mutant sequences. Conformational changes were predicted for mutant *hsa-miR-622*, *hsa-miR-767-5p* and *hsa-miR-1280* (Figure S1). However, conformational changes predicted *in silico* rarely equate to a physiological effect [12] and the functional implication of mutations identified here requires further investigation with *in vitro* assays.

In contrast to the frequent observation of tumor-specific variations contributing to the activation or repression of protein coding genes in cancer, these findings demonstrate that somatic mutations in miRNA genes are an infrequent event during ovarian pathogenesis and add to accumulating evidence from a range of tumor types suggesting that miRNAs are rarely dysregulated by this mechanism. Given the large number of mRNA targets predicted for a single miRNA and the diverse roles of those predicted target genes, any somatic mutation in a miRNA gene (and particularly those occurring within the seed sequence) are likely to impact many biological pathways [2], [3], some of which may be involved in maintaining cell homeostasis. Consequently, even if a somatically mutated miRNA gene altered mRNA expression to positively affect tumor survival, it is likely that there would be a larger number of gene expression changes which would not be conducive to tumor cell survival. Conversely, in certain situations, mRNA transcriptional repression may result from the action of multiple miRNAs [28] and as such, the altered activity of a single miRNA may be insufficient to result in a biological effect. Finally, it is becoming increasingly recognised that miRNA alterations observed in cancer tissues may occur secondary to defects in components of the miRNA processing machinery, including transcription factors and chromatin remodelling genes regulating miRNA transcription, as well as components of miRNA post-transcriptional regulation [29], [30]. In ovarian tumors, *DICER1* and *EIF2C2* (Argonaute2) DNA copy number gains have been observed in 24.5% and 51.5% of tumors respectively [9] and median overall survival is reduced among women whose tumors have lower *DICER1* and *DROSHA* mRNA expression [31]. Further investigation is needed establish the importance of alterations to these and other components of the miRNA biogenesis pathway in the pathogenesis of ovarian cancer.

### Somatic mutations targeting 3′-untranslated regions are infrequent events in ovarian tumors

To investigate the possibility that somatic mutations occurring within the 3′-UTRs of cancer genes alter an existing miRNA binding site, and thus signify a novel mechanism for aberrant mRNA gene expression in cancer, we sequenced the 3′-UTRs of 11 oncogenes (*AKT2*, *BRAF*, *CCNE1*, *CTNNB1*, *EGFR*, *ERBB2*, *FGF1*, *KRAS*, *MYC*, *PIK3CA* and *RAB25*) by targeted re-sequencing in the 86 ovarian tumors. In the case of an oncogene, abrogation of a miRNA binding site may permit unregulated oncogene expression. In addition, the 3′-UTRs of 7 tumor suppressor genes (TSGs) (*BRCA1*, *BRCA2*, *CDKN2A*, *PTEN*, *RB1*, *SPARC* and *TP53*) were also sequenced to assess the prevalence of somatic mutations that would generate a *de novo* miRNA binding site and result in TSG down-regulation. These genes are recognized by the Cancer Gene Census to be causally implicated in ovarian tumour development either by somatic mutation, gene amplification or deletion [32]. *FGF1*, *RAB25* and *SPARC* were chosen based on their demonstrated functional role in ovarian cancer [33]–[37]. 3′-UTRs were sequenced to 108-fold mean coverage and greater than 99% of the targeted bases within these regions were covered by 10 or more sequence reads. Tumor-specific mutations were infrequently identified in the 3′-UTRs of ovarian samples: 4/86 tumor samples were each identified with a mutation in one of four different oncogenes (*AKT2*, *CTNNB1*, *EGFR* and *ERBB2*) (Table 1), with no mutations detected in the 7 TSGs investigated. *In silico* miRNA target prediction algorithms suggest that mutated loci in *AKT2*, *CTNNB1* and *ERBB2* may occur within the region of a predicted miRNA binding site, with two mutations, the c.*538T>A (*CTNNB1*) and c.*460G>C (*ERBB2*) substitutions, predicted to occur within the seed sequence of *hsa-miR-630* and *hsa-miR-640* binding respectively. RNA expression profiling was available for samples with mutations in *AKT2* and *EGFR* and demonstrated that the transcript levels of these genes was not altered in samples with somatic 3′-UTR mutations relative to other tumor samples of the same ovarian subtype (Figure S2).

**Table 1:** Somatic mutations identified in 3′-untranslated regions of candidate mRNAs.

Gene	Nucleotide change^1^	MicroRNA binding affected by mutation	Predictive algorithm	Sample ID
*AKT2*	c.*892C>T	*hsa-miR-429*	DIANA-microT [47], [48]	P1768
*CTNNB1*	c.*538T>A	*hsa-miR-640* 2	miRanda [49]	P0511
		*hsa-miR-10a*	miRanda	
		*hsa-miR-587*	miRanda	
*EGFR*	c.*101C>G	-	-	IC151
*ERBB2*	c.*460G>C	*hsa-miR-495*	miRanda	P5514
		*hsa-miR-630* 2	microCOSM Targets [40]	

1 Nucleotide changes are described relative to the following sequences: *AKT2* NM_001626.3; *CTNNB1* NM_001904.3; *EGFR* NM_005228.3; *ERBB2* NM_004448.2.

2 Indicates that the somatic mutation occurs within the microRNA-mRNA interaction at the seed region.

Although it is recognised that miRNAs can also impart transcriptional repression through action on the 5′-UTR of an mRNA target, this study provides preliminary evidence that somatic mutations altering miRNA binding sites within the 3′-UTR of common cancer genes are infrequent in epithelial ovarian tumors. Effective translational silencing may require synergistic action of miRNAs at multiple sites across a UTR, either by a single family of microRNAs or by a combination of unrelated microRNAs, and thus the single somatic mutations identified here are likely insufficient to silence the respective transcript [28], [38]. The somatic mutation prevalence in 3′-UTR regions of candidate cancer genes sequenced was 1.43 mutations per Mb. By comparison, the mutation prevalence in protein coding regions of known cancer genes in this tumor cohort is 570.57, 183.60 and 32.63 mutations per Mb for *TP53*, *KRAS* and *PIK3CA* respectively, while the estimated mutation prevalence in the coding exome is 2.4 mutations per Mb in ovarian tumors [23]. Thus, it is likely that the low rate of mutation in 3′UTRs compared to exons indicates that the majority of mutations in 3′-regulatory regions identified here occur as bystander events in tumor cell development.

In summary, somatic mutations in miRNA genes were infrequently observed in ovarian tumors and thus are unlikely to account for altered miRNA activity observed in this tumor type. In addition, we provide preliminary evidence that selection for somatic mutations within the 3′-UTRs of candidate cancer genes, which would be hypothesised to interfere with miRNA dependent gene regulation, is unlikely to represent a common mechanism for altered mRNA expression in ovarian tumors.

## Materials and Methods

### Ethics statement

Accrual and use of patient material for this study was approved by the following Human Research Ethics Committees: Southampton Hospital Human Research Ethics Committee, Peter MacCallum Cancer Centre Human Research Ethics Committee, Queensland Institute of Medical Research Human Research Ethics Committee, University of Melbourne Human Research Ethics Committee, Westmead Hospital Human Research Ethics Committee. All individuals gave written informed consent for the use of their tissue in research. This project was approved by the Peter MacCallum Cancer Centre Human Research Ethics Committee (Approval # 09/29).

### Ovarian tumor cohort

86 primary epithelial ovarian tumor tissue samples were obtained through the Peter MacCallum Cancer Centre Tissue Bank, Australia Ovarian Cancer Study or from patients presenting to hospitals in the south of England [39]. All tumor DNA samples were microdissected to ensure greater than 80% epithelial cell component. This tumor cohort comprised a mixture of serous (n = 45), endometrioid (n = 28), mucinous (n = 7) and clear cell (n = 6) subtypes. Matching peripheral blood samples were also collected from all patients at time of tumor collection and used as a source of germline DNA.

### Candidate region identification for targeted next-generation sequencing

The 3′-UTRs of 18 protein coding genes were selected for somatic mutation screening. Genome co-ordinates for selected 3′-UTRs were identified based on those annotated in the Ensembl database (release 54) for the following transcripts: *AKT2* (ENST00000392038), *BRAF* (ENST00000288602), *CCNE1* (ENST00000262643), *CTNNB1* (ENST00000349496), *EGFR* (ENST00000275493 and ENST00000344576), *ERBB2* (ENST00000269571), *FGF1* (ENST00000359370), *KRAS* (ENST00000256078), *MYC* (ENST00000259523 and ENST00000377970), *PIK3CA* (ENST00000263967), *RAB25* (ENST00000361084), *BRCA1* (ENST00000309486), *BRCA2* (ENST00000380152), *CDKN2A* (ENST00000304494), *PTEN* (ENST00000371953), *RB1* (ENST00000267163), *SPARC* (ENST00000231061) and *TP53* (ENST00000269305). The genomic co-ordinates for human precursor miRNAs were obtained from the Sanger Institute miRBase (release 13.0, March 2009) [40], [41], including 548 individual miRNA genes as well as 164 miRNAs within 62 miRNA clusters. All coding exons of *TP53*, *KRAS* and *PIK3CA* were included for sequence analysis.

### Library preparation and target enrichment

200 ng of tumor or matched normal lymphocyte DNA was randomly fragmented to approximately 200 bp (Covaris, Woburn, MA) and end repair and A-tailing performed according to the Illumina genomic DNA library preparation protocol (Illumina, San Diego, CA). Following this, DNA was ligated with one of 7 custom multiplexing adapters compatible with Illumina single end sequencing. Indexed DNA samples were pooled equally prior to PCR enrichment. All reagents used during library preparation were obtained from New England Biolabs (NEB, Ipswich, MA). A boutique exon capture (SureSelect, Agilent Technologies, Santa Clara, CA) was used to specifically enrich for selected 3′-UTRs, miRNAs and coding exons of cancer genes from genomic DNA libraries prior to next generation sequencing. Capture probes were designed using default parameters by submitting genomic co-ordinates to eArray (Agilent Technologies, Santa Clara, CA). Solution hybridization, washing, elution and amplification were performed according to the recommended protocol.

### Somatic mutation analysis by Illumina GAIIx sequencing and capillary electrophoresis

Target enriched DNA libraries were sequenced on an Illumina GAIIx, generating 75 bp single end sequence reads. Image analysis and base calling was performed using the Genome Analyser Pipeline v1.6. Sequence reads were aligned to the human reference genome (GRCh37/hg19 assembly) using BWA and remaining unmapped reads were aligned with Novoalign software [42]. This was followed by local realignment with GATK [43]. Point mutations and insertions/deletions (indels) were identified using GATK and Dindel [44] respectively and annotated with information from Ensembl release 56. Only mutations within miRNA transcripts annotated in miRBase were considered for further analysis.

Point mutations and indels were identified as somatic alterations only when (*i*) the variant was not called in the matched normal sample or identified as a germline alteration in another tumor/normal pair (*ii*) the variant was not seen in >2% of reads in the matched normal sample following manual inspection of sequence reads using the Integrated Genomics Viewer [45] (*iii*) the variant was identified in at least four unique sequence reads with at least two mapping in the forward and two mapping in the reverse orientation.

All mutations which met the above criteria were subjected to validation by conventional PCR amplification and bidirectional capillary electrophoresis on the ABI3130 Genetic Analyser using BigDye Terminator v3.1 sequencing chemistry (Applied Biosystems, Foster City, CA).

### Identification of miRNA-binding sites

The TargetScan (release 5.2) [46], microCOSM Targets (version 5) [40], DIANA-MicroT (version 3.0) [47], [48] and miRanda (release August 2010) [49] predictive algorithms were used to determine whether the somatic mutations detected in mRNA 3′UTRs occurred within miRNA binding sites. A mirSVR score threshold of less than −0.1 and minimum folding energy score threshold of less than or equal to −16 kcal/mol were used for the miRanda algorithm. Default parameters were used for all other algorithms.

## Supporting Information

Figure S1 **Predicted secondary structure changes as a result of somatic mutations in miRNA transcripts.** Mature sequences are shadowed and the mutated base indicated by the arrowhead in (**a**) *hsa-miR-622*, (**b**) *hsa-miR-1280* and (**c**) *hsa-miR-767-5p*. The precursor miRNA sequence plus 50 bp flanking the precursor at the 5′ and 3′ ends was used to predict the secondary structure with the lowest free energy by the RNAfold program [29] using default parameters.
(PDF)

Figure S2 ***AKT2*** **and** ***EGFR*** **mRNA expression is not altered in the presence of 3′-untranslated region somatic mutations relative to other ovarian samples of the same subtype**. (**a**) *AKT2* expression in endometrioid tumors, including sample P1768 with an *AKT2* c.*892C>T somatic mutation (indicated in red). mRNA expression profiling data was obtained from Tothill *et al.* [50]. *AKT2* expression probe sets 225471_s_at and 226156_at are shown. (**b**) *EGFR* expression in endometrioid tumors, including sample IC151 with an *EGFR* c.*101C>G somatic mutation (indicated in red). mRNA expression profiling data was obtained from Ramakrishna *et al.* [51]. Error bars are representative of mean ± SD.
(PDF)

Table S1 **Clinical characteristics of ovarian tumors sequenced for somatic mutations in microRNA genes and candidate 3′-untranslated regions.**
(XLS)
