# Supplementary figures and images for: MicroRNA Genes and Their Target 3′-Untranslated Regions Are Infrequently Somatically Mutated in Ovarian Cancers
Source: PLoS One. 2012 Apr 20;7(4):e35805. doi: 10.1371/journal.pone.0035805 (PMC3334977; doi:10.1371/journal.pone.0035805)

Figure S1

A

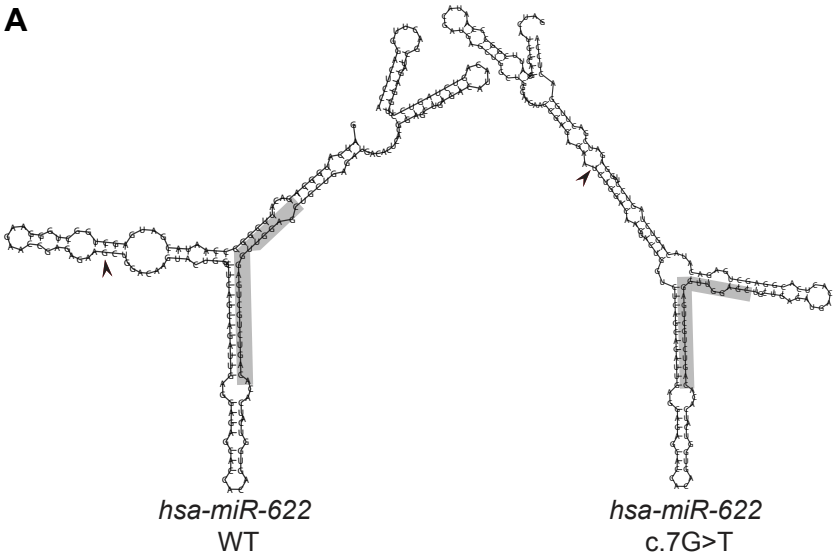

B

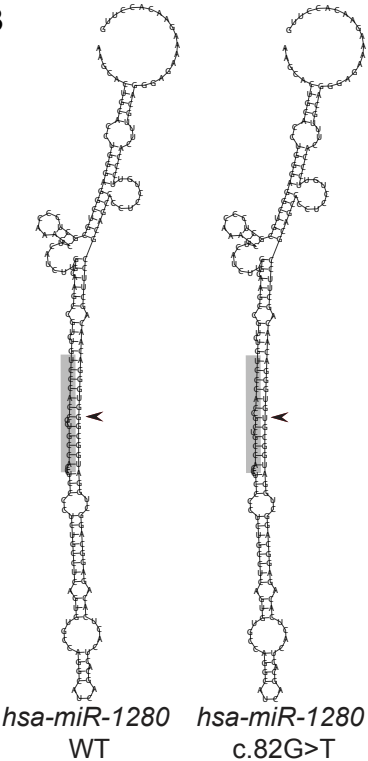

C

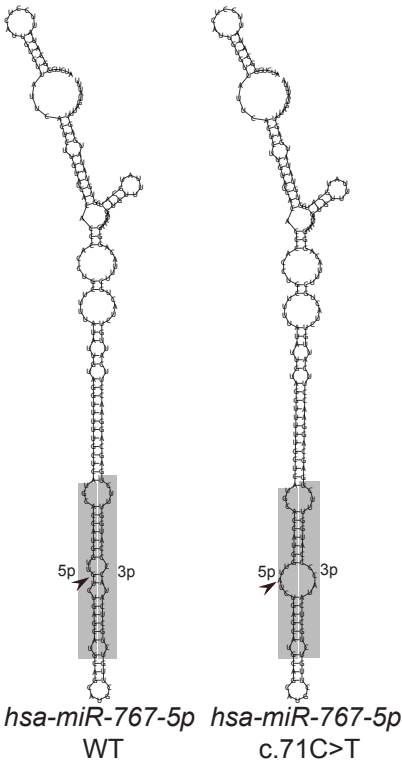

Supplement: Figure S1 — Predicted secondary structure changes as a result of somatic mutations in miRNA transcripts. Mature sequences are shadowed and the mutated base indicated by the arrowhead in (a) hsa-miR-622, (b) hsa-miR-1280 and (c) hsa-miR-767-5p. The precursor miRNA sequence plus 50 bp flanking the precursor at the 5′ and 3′ ends was used to predict the secondary structure with the lowest free energy by the RNAfold program [29] using default parameters. (PDF) [file pone.0035805.s001.pdf]

**Figure S2**

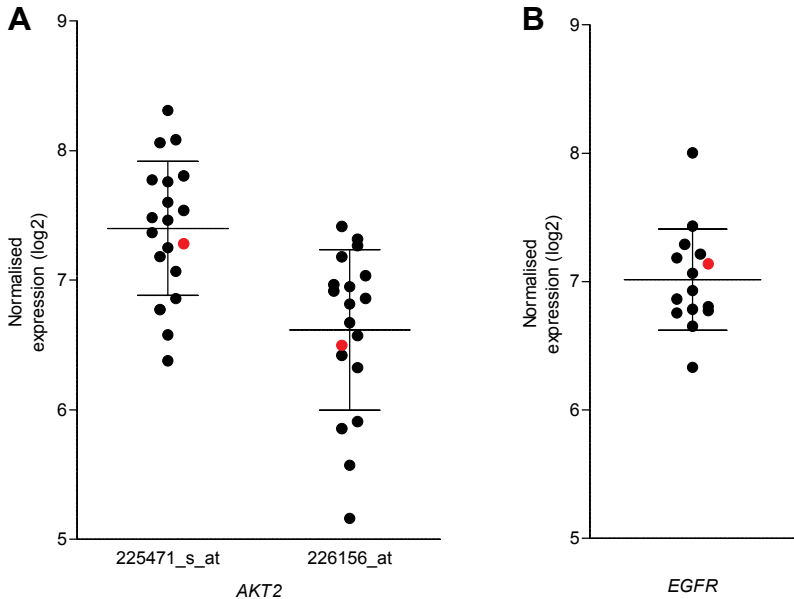

Supplement: Figure S2 — AKT2 and EGFR mRNA expression is not altered in the presence of 3′-untranslated region somatic mutations relative to other ovarian samples of the same subtype. (a) AKT2 expression in endometrioid tumors, including sample P1768 with an AKT2 c.*892C>T somatic mutation (indicated in red). mRNA expression profiling data was obtained from Tothill et al. [50]. AKT2 expression probe sets 225471_s_at and 226156_at are shown. (b) EGFR expression in endometrioid tumors, including sample IC151 with an EGFR c.*101C>G somatic mutation (indicated in red). mRNA expression profiling data was obtained from Ramakrishna et al. [51]. Error bars are representative of mean ± SD. (PDF) [file pone.0035805.s002.pdf]
